# Challenging the assumption that experience- and training-related factors improve accuracy of cervical cancer screening image interpretation: Evidence from Tanzania

**DOI:** 10.1371/journal.pgph.0007074

**Published:** 2026-08-10

**Authors:** Federica Inturrisi, Safina Yuma, Bariki Mchome, Alex Mremi, Prisca Dominic Marandu, Nicola West, Melinda Chelva, Rohini R. Datta, Amber Simpson, Kayode O. Ajenifuja, Clement A. Adepiti, Kanan T. Desai, Nicolas Wentzensen, Jennifer Boyd-Morin, Didem Egemen, Jose A. Jeronimo, Silvia de Sanjosé, Mark Schiffman, Karen Yeates

**Affiliations:** 1 National Cancer Institute, Rockville, Maryland, United States of America; 2 MSF Foundation, Paris, France; 3 Ministry of Health, Community Development, Gender, Elderly and Children, Dodoma, Tanzania; 4 Kilimanjaro Clinical Research Institute, Moshi, Tanzania; 5 KCMC University, Moshi, Tanzania; 6 Pamoja Tunaweza Women’s Centre, Moshi, Tanzania; 7 Queen’s University, Kingston, Canada; 8 Obafemi Awolowo University, Ile-Ife, Nigeria; 9 Norwalk Hospital Internal Medicine Residency Program, Norwalk, Connecticut, United States of America; 10 Information Management Services Inc, Calverton, Maryland, United States of America; 11 ISGlobal, Barcelona, Spain; PLOS: Public Library of Science, UNITED STATES OF AMERICA

## Abstract

Visual inspection of the cervix with acetic acid (VIA), although recognized as inaccurate, remains widely used for cervical screening in low-resource settings. Training and image review by more experienced providers − such as the smartphone-enhanced VIA (SEVIA) in Tanzania − are assumed to improve VIA performance, but remain untested against a reference standard of disease. Between June and December 2023, 486 VIA providers in Tanzania completed a questionnaire on experience- and training-related factors (years practicing VIA, patient volume, refresher training, SEVIA training) and independently reviewed a set of 50 high-quality cervical images (24,300 total reviews). Image reference diagnosis was defined using HPV status and histology: precancer if HPV-positive and histologically-confirmed CIN3, normal if HPV-negative and normal histology, otherwise indeterminate. For comparability, providers’ image reviews were recategorized into positive (VIA-positive for ablation, LEEP, or suspect cancer) or negative (VIA-negative). In an image-level analysis, we calculated proportion of providers labeling each image as positive and averaged those within subgroups of experience/training and reference diagnosis. Overall, providers (mean VIA experience 3.7 years, weekly VIA exams 12.2) showed moderate sensitivity: on average 60.0% (95% confidence interval 59.2 − 60.7) labeled precancers as positive and 54.4% (53.0 − 55.9) labeled normals as negative. More experienced providers tended to be more specific but less sensitive: for example, an average of 56.5% (55.2–57.9) of providers with ≥5 years of VIA experience labeled precancers as positive vs 61.4% (60.5–62.4) of providers with <5 years’ experience; corresponding proportions for images with normal diagnoses were 36.8% (34.3–39.4) vs 44.5% (42.7–46.3). In this image-based evaluation using a virologic/histologic reference diagnosis, VIA showed moderate sensitivity for detecting cervical precancer, and greater provider experience did not substantially increase overall accuracy. These findings challenge assumptions that experience alone improves VIA diagnostic performance, highlighting the need for objective quality assurance and more accurate screening methods.

## Introduction

Cervical cancer remains one the most common cancers in women worldwide despite the existence of effective preventive measures including prophylactic vaccination against the causative agent human papillomavirus (HPV) and HPV-based screening for precancerous lesions of the cervix. The burden of disease is highest in low- and middle-income countries (LMICs), where organized prevention programs tend to be limited. Despite the recommendations to screen using HPV testing due to its high negative predictive value and reproducibility [1], few LMICs have introduced or have imminent plans to introduce HPV DNA testing [2]. The most widely used screening method in LMICs, often offered opportunistically and with low coverage, is visual inspection of the cervix with acetic acid (VIA). VIA has been used for a few decades as it is a simple and inexpensive method that relies on health care providers visually identifying, by naked eye, acetowhite areas at the cervical transformation zone appearing one minute after the application of 3–5% acetic acid solution. However, VIA is inaccurate in distinguishing cervical precancer from common minor abnormalities, leading to both overtreatment and undertreatment [3–8].

Over the years, efforts have been made to improve performance of VIA. Numerous interventions exist, focusing on both extensive/refresher training of health care providers by more experienced providers and on the development of mobile health (mHealth) and telemedicine tools allowing capturing, sharing and reviews of cervical images by experienced providers (aka ‘reviewers’). Examples include the smartphone-enhanced VIA (SEVIA) in Tanzania [9,10], the APIN-developed VIA visual application (AVIVA) in Nigeria [11], and the electronic cervical cancer control (eC3) technology in Zambia [12], all based on training, mentorship and review of cervical images that were introduced as part of the cervical screening programs and used to scale up VIA services in the respective countries. Together with standardized components of image collection, the use of those and similar tools assumes that the accuracy of visual evaluation is higher when performed by highly trained/experienced providers as compared to less trained/experienced providers. However, such assumption has never been tested. While tens of thousands of women have been screened, such interventions mainly showed improved ‘concordance’ in VIA diagnosis between trainees and mentors (aka ‘providers and reviewers’), but no comparison of VIA accuracy with disease reference standard (i.e., histologic diagnosis) was made.

We assessed whether providers with more experience and training in VIA have greater concordance with the reference standard when reviewing digital cervical images compared to less experienced providers. Health care providers from Tanzania’s national VIA screening program reviewed a set of digital cervical images with known virologic/histologic diagnosis.

## Materials and Methods

### Ethics statement

This study was approved by the Tanzania National Institute for Medical Research (NIMR; protocol number NIMR/HQ/R.8a/Vol.IX/4284) and by Kilimanjaro Christian Medical University College (KCMUCo; clearance number 2623) (clinical trial number: not applicable). The study was conducted in accordance with the principles of the Declaration of Helsinki. All study participants provided written informed consent. Consenting procedures, study instructions, and the image review sessions were delivered in Swahili to ensure clear understanding and consistency across all participating providers. Additional information regarding the ethical, cultural, and scientific considerations specific to inclusivity in global research is included in the Supporting Information (**Checklist A in** **S1 Appendix**).

### Image set and reference diagnosis

The image set included 50 cervical digital images of women with a mean age of 35 years (range 25–46) who participated in prior studies led by the US National Cancer Institute (NCI): 27 images from the Biopsy Study and 23 images from Project Itoju.

The design of those has been published previously [13–15]. In brief, the Biopsy Study is a population-based study of women 18 years and older who were referred for colposcopy at the University of Oklahoma colposcopy clinic in 2009–2011 for screening results of HPV-positive atypical squamous cells of undetermined significance, low-grade squamous intraepithelial lesion (ASC-US/LSIL), or high-grade squamous intraepithelial lesion (HSIL) independent of HPV status. Exclusion criteria were previous treatment for cervical disease (ablative treatment, excisional treatment, hysterectomy, chemotherapy or radiation), pregnancy, and HIV infection. All women enrolled in the study had a colposcopy conducted and at least one biopsy taken (up to a maximum of four), with digital photographic documentation of biopsy sites. Images were acquired using a Nikon D700 camera with a beam splitter attached to a Zeiss FC150 colposcope. Before colposcopic biopsy procedures, a cervical cytology specimen was collected and used for ThinPrep liquid-based cytology, p16/Ki-67 immunostaining, and HPV DNA analysis. HPV testing was done using the Linear Array HPV Genotyping Test (Roche Molecular Diagnostics).

Project Itoju is a series of population-based screening studies in rural Nigeria, beginning in 2009–2010 [16]. The images and data used in this study came from the third phase in 2018–2020 in Ile-Ife [15]. Non-pregnant women of 30–49 years were invited by a public message campaign to collect a cervico-vaginal sample for HPV testing using a self-collection kit with a cervical brush placed into specimen transport medium (STM) tube (Qiagen). HPV testing was done with the Hybrid Capture-2 HPV DNA Test (Qiagen). Women with a positive HPV result were referred for a colposcopy visit. Those with a history of cervical cancer, hysterectomy, or pregnant at the time of the visit were excluded. During standard colposcopy examination, up to a maximum of four biopsies were collected. Prior to colposcopic biopsy procedures, cervical images were taken with three different devices: a Samsung Galaxy S8 smartphone, a MobileODT EVA device, and a Zeiss FC150 colposcopic image captured via a beam splitter by a DSLR camera.

For the image set used in this study, one image per woman was selected. The selection of images from Project Itoju was limited to colposcopic images, to match the Biopsy Study collection. All images had HPV and histopathology results; this specific set was prioritized for the rigor of its reference diagnosis. At the time of this study, a comparable Tanzanian image bank with high-quality digital images and verified HPV and histopathology was not available.

Histologic interpretation of both studies was initially conducted using cervical intraepithelial neoplasia (CIN) terminologies, which we translated to obtained a more definite case-control distinction. Specifically for this analysis, the reference diagnosis was stringently defined using a combination of virologic and histologic results: images from women with HPV-positive status and histologically-confirmed CIN3 were labeled as precancer; images from women with HPV-negative status and normal histology were labeled as normal; all other images (i.e., HPV-positive status and normal/low-grade/CIN2 histology) were labeled as indeterminate. The latter indeterminate category aims to capture ambiguous visual profiles or discordant results due to the proven poor reproducibility of diagnosis of low-grade and CIN2 lesions. Hereafter, we will refer to these as reference diagnosis categories (precancer, normal, or indeterminate). Images of precancers and indeterminate were similarly distributed between the two studies; images of normals came from the Biopsy study due to the design of the Project Itoju where only HPV-positives were referred for colposcopy, images and biopsies.

Selection for the 50-image set was done by stratified random selection to include a high proportion of cases: 26 (52.0%) precancers, 8 (16.0%) normals, and 16 (32.0%) indeterminate. Images had previously been reviewed by the NCI for image quality and images with low quality were excluded. One evaluable image was then selected at random for each woman. All selected images met pre-defined quality standards. They consisted of high-resolution digital colposcopic color images captured at a standardized density of 300 dpi. Depending on the original study repository, image dimensions ranged from 4256x2832 pixels (average file size ~2 MB) to 6016x4016 pixels (average file size ~12 MB), with an overall average file size of 7 MB per image across the 50-image set. In all images, the cervix occupied the majority of the frame under standard low-magnification settings.

Images were provided anonymized and unlabeled when the health care providers reviewed them for visual impressions during the study periods in Tanzania.

### Study population and design

Health care providers of the Tanzania’s national Cervical Cancer Prevention (CECAP) Program were invited to participate during routine training/supervision sessions coordinated by Ministry of Health (MoH) implementation partners (Elizabeth Glaser Pediatric AIDS Foundation (EGPAF) and Management and Development for Health (MDH)) across eight regions (Arusha, Dodoma, Kilimanjaro, Manyara, Singida, Dar es Salaam, Kagera, and Tabora). Participation to the study was offered to all providers attending the EGPAF and MDH events. The target sample size was 400–500 CECAP providers to review the 50-image set, ensuring a sufficiently large number of individual reviews to support stratified analyses across key experience measures. Between 19 June and 18 December 2023, 486 providers consented, completed a short questionnaire capturing experience- and training-related factors (job title, years of VIA experience, average number of VIA exams performed weekly, receipt of VIA refresher training and/or SEVIA training) and independently reviewed the 50-image set.

Reviews of unlabeled (i.e., masked reference diagnosis) images were performed in groups but under supervision to guarantee silent conditions and independent assessment. Images were displayed for 45 seconds with a 15-second break between images; providers recorded one of the following categories per image: ungradable (i.e., image quality is ungradable/cannot perform VIA on this image), VIA-negative, VIA-positive and eligible for ablation, VIA-positive requiring loop electrosurgical excision procedure (LEEP, also known as LLETZ), or suspect cancer (referral/biopsy). The approach and set-up were very similar to those used by the implementation partners during CECAP training and were discussed in advance with key persons of the MoH, EGPAF, and MDH.

### Data analyses

The analysis aimed to assess whether provider experience- and training-related factors are associated with accuracy in visual interpretation of cervical images.

For comparability with the reference diagnosis, we dichotomized reviews into positive (VIA-positive including those with recommendations of ablation, LEEP, or suspect cancer) and negative (VIA-negative) for precancer. Reviews classified as ungradable (1,330 in total) were treated as missing for that reviewer.

Experience and training variables were categorized as: years of VIA experience (tertiles, grouped as 3^rd^ or most experienced vs 1^st^ & 2^nd^; later reclassified as ≥5 years vs < 5 years), number of VIA exams performed weekly (tertiles, grouped as 3^rd^ or most experienced vs 1^st^ & 2^nd^), receipt of VIA refresher training (yes vs no), receipt of SEVIA training (yes vs no). Additionally, an ‘expert group’ was defined within the study pool as providers who fell into the highest tertile for both years of experience and weekly exam volume, and who had also received both refresher and SEVIA training. This composite group was constructed to avoid collinearity between single experience and training variables, while identifying a restricted subset of individuals representing the highest combination of these metrics. By grouping these highly experienced providers, we aimed to evaluate whether this specific subset demonstrated superior visual evaluation accuracy compared to the rest of the cohort.

We conducted image-level analyses by experience/training variables and overall, separately for each reference diagnosis category. The dataset underlying these image-level analyses is provided in the Supporting Information (**Table A in** **S1 Appendix**). Since each image was evaluated by all 486 providers, we calculated the proportion of providers who labeled each image as positive. In order to compare different experience/training levels, we averaged those proportions within each experience/training subgroup and diagnosis category. We present proportions with 95% confidence intervals (CIs) computed via normal approximation, and note where CIs do or do not overlap. Within each diagnosis category, for each image we assumed independent and non-identical binomial distributions (i.e., the probability of a positive review for each image is different). We also assumed that providers with similar experience/training levels had the same probability of labeling a certain image as positive.

If the hypothesis holds that more experienced providers are overall more accurate in visual evaluation of cervical images, we expect providers within each subgroup of higher experience/training category (i.e., number of years of VIA experience – tertile 3, number of VIA exams performed weekly – tertile 3, received VIA refresher training – yes, received SEVIA training – yes, expert group – yes) to yield larger proportions of positive reviews among precancers (i.e., higher sensitivity) and smaller proportions of positive reviews among normals (i.e., higher specificity).

Analyses were completed for all providers and repeated for subgroups defined by job title, restricting only to nurses or only to doctors.

## Results

### Characteristics of providers and image reviews

A total of 486 CECAP providers participated across 8 regions in Tanzania. The study population is described in **Table 1**. Most were nurses (n = 416; 85.6%); 60 (12.3%) were other medical professionals (medical doctors, clinical officers, health practitioners, or gynecologists), and 10 (2.1%) indicated other or none of the listed professions. On average, providers had 3.7 years of VIA experience (n = 484 reported; 99.6%). The average number of VIA exams performed weekly was 12.2 (n = 477 reported; 98.1%). Details on the tertiles distribution of years of VIA experience and weekly VIA exams are given (Table 1). Two providers (0.4%) reported having only on-the-job training without formal training. Among those who reported having received VIA refresher training (n = 237 reported; 48.8%), the mean time since training was 1.9 years (n = 192 reported; 81.0%). Among those who reported having received SEVIA training (n = 63 reported; 13.0%), the mean time since training was 2.5 years (n = 55 reported; 87.3%).

**Table 1:** Characteristics of health care providers and reviews of the 50-image set.

	N (%)	Mean (SD)	Median (range)
Total number of providers	486 (100.0)		
**Region in Tanzania**			
Arusha	30 (6.2)		
Dar es Salaam	131 (27.0)		
Dodoma	37 (7.6)		
Kagera	133 (27.4)		
Kilimanjaro	32 (6.6)		
Manyara	27 (5.6)		
Singida	38 (7.8)		
Tabora	58 (11.9)		
**Job title**			
Nurse	416 (85.6)		
Doctor	60 (12.3)		
Other	8 (1.6)		
Missing	2 (0.4)		
**Years of VIA experience**			
Missing	2 (0.4)		
Non-missing	484 (99.6)	3.7 (3.3)	3.0 (0.1-35.0)
[if non-missing] Tertile distribution			
Tertile 3	150 (31.0)	7.5 (3.5)	7.0 (5.0-35.0)
Tertile 1&2	334 (69.0)	1.9 (1.1)	2.0 (0.1-4.0)
Tertile 2	104 (21.5)	3.3 (0.5)	3.0 (3.0-4.0)
Tertile 1	230 (47.5)	1.3 (0.6)	1.0 (0.1-2.0)
**Number of VIA exams performed weekly**			
Missing	9 (1.9)		
Non-missing	477 (98.1)	12.2 (13.9)	8.0 (1.0-100.0)
[if non-missing] Tertile distribution			
Tertile 3	159 (33.3)	25.7 (17.1)	20.0 (12.0-100.0)
Tertile 1&2	318 (66.7)	5.5 (2.7)	5.0 (1.0-11.0)
Tertile 2	122 (25.6)	8.5 (1.7)	9.0 (6.0-11.0)
Tertile 1	196 (41.1)	3.7 (1.2)	4.0 (1.0-5.0)
**VIA refresher training**			
Missing	3 (0.6)		
On-the-job training	2 (0.4)		
No	244 (50.2)		
Yes	237 (48.8)		
[if yes] Time since training (years)	192 (81.0)	1.9 (2.1)	1.0 (0.0-17.0)
**SEVIA training**			
Missing	1 (0.2)		
No	422 (86.8)		
Yes	63 (13.0)		
[if yes] Time since training (years)	55 (87.3)	2.5 (2.6)	1.0 (0.0-7.0)
Total number of reviews	24,300 (100.0)		
**Providers’ reviews**			
Missing/Ungradable	1,349 (5.6)		
Positive	11,603 (47.7)		
Negative	11,348 (46.7)		

Abbreviations: SD, standard deviation; SEVIA, smartphone-enhanced VIA; VIA, visual inspection with acetic acid.

Across 486 providers and 50 images there were 24,300 reviews, representing the image-level dataset (**Table 1**). There were 19 (0.1%) missing reviews and 1,330 (5.5%) ungradable reviews, given by 29 (6.0%) providers and corresponding to 574 (43.2%) precancers, 627 (47.1%) indeterminate, and 129 (9.7%) normals. Of the 11,603 (47.7%) positive reviews, the majority were precancers (65.3%), but 14.1% were normal diagnoses. Of the 11,348 (46.7%) negative reviews, 39.4% were precancers and only 18.7% were normal diagnoses. Indeterminate diagnoses were labeled positive in 30.7% of reviews and negative in 61.2%.

### Impact of provider experience- and training-related factors on image evaluation

The overall agreement among provider reviews was 60.0% (95% CI 59.2 − 60.7) for precancers and 54.4% (95% CI 53.0 − 55.9) for normals.

**Fig 1:**
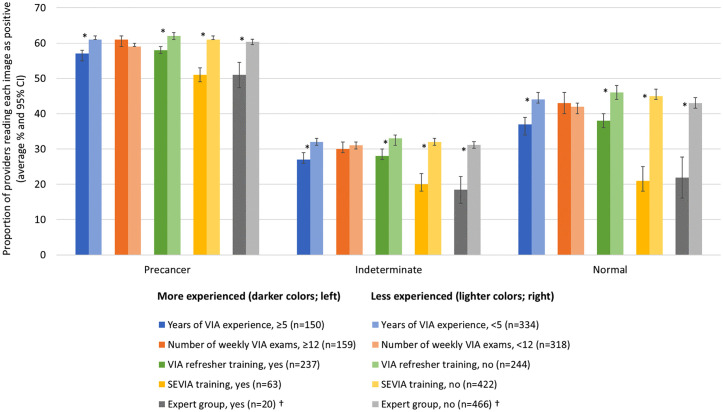
Average proportions of providers reading each image as positive by reference diagnosis and experience/training subgroups. * Pairwise comparisons with non-overlapping CIs. † Based on a combination of the four experience variables: ‘Expert group, yes’ (n= 20) were providers that had  ≥ 5 years of VIA experience, performed ≥12 weekly VIA exams, received VIA refresher training, and received SEVIA training; ‘Expert group, no’ (n= 466) were all other providers. Abbreviations: CI, confidence interval; SD, standard deviation; SEVIA, smartphone-enhanced VIA; VIA, visual inspection with acetic acid.

Fig 1 summarizes the average proportion of providers labeling each image as positive in higher vs lower experience/training subgroups (years of experience, weekly volume, refresher training, SEVIA training, and composite expert group), separately by reference diagnosis category.

On average, providers with greater experience (e.g., more years of experience, refresher and/or SEVIA training) gave fewer positive reviews across all categories of reference diagnosis, consistent with higher specificity but lower sensitivity compared with less experienced providers.

For example, the average proportion of providers giving a positive review to an image with precancer diagnosis was 56.5% (95% CI 55.2–57.9) among those with ≥5 years of VIA experience vs 61.4% (95% CI 60.5–62.4) among those with <5 years of experience, indicating that providers with more VIA experience had lower sensitivity in detecting precancers. The corresponding average proportions of providers giving a positive review to an image with normal diagnosis were 36.8% (95% CI 34.3–39.4) vs 44.5% (95% CI 42.7–46.3) (Fig 1**; Table B in** S1 Appendix), respectively, indicating that providers with more VIA experience had a higher specificity in reviewing normals.

Differences by experience/training category were statistically significant (average proportions with non-overlapping 95% CI) for years of VIA experience, refresher training, SEVIA training, and the composite expert grouping; they were not significant for the number of VIA exams performed weekly. Results were similar when restricting analyses to nurses only or doctors only.

Figures for positive/negative reviews by precancer/normal diagnoses are also given in the Supporting Information (**Table B in** S1 Appendix) stratified by experience/training subgroup.

## Discussion

We evaluated the clinical accuracy of VIA providers, using Tanzania’s national VIA screening program as an example, in visual impressions of enlarged digital images pre-selected for high quality and to contain a high number of precancers. This is not a complete evaluation of VIA. This study focuses on reviews of digital cervical images by provider experience- and training-related factors as a component of the SEVIA and other programs used to support VIA screening programs [17]. Our results showed an overall moderate sensitivity of visual diagnosis of CIN2+ and no strong evidence that accuracy improves by increasing years of experience or training sessions in visual diagnosis of cervical images. This study therefore challenges the assumption that experience and training lead reliably to higher performance of visual evaluation methods for cervical screening.

Over the last decade, the use of mHealth in cervical screening in LMICs has been brought forward as a tool to bring quality assurance to VIA through different implementation methods, and to therefore improve performance of VIA programs, either in primary or triage settings. This includes opportunities for providers to receive repetitive ‘supportive supervision’ or refresher training through virtual methods or asynchronous review of digital images (i.e., online tutorials using cervical image review) with more experienced trainers/providers. There are several examples of those mHealth tools in LMICs, allowing for training, supervision, peer review, quality assurance, and expert opinion through immediate distance consultation, if needed [12]. One of those is SEVIA, used in several regions in Tanzania since 2016 to improve VIA quality and to evaluate the impact of a quality assurance program within the national CECAP program. An important component of quality control processes of SEVIA is the ‘co-assessment’ and supportive supervision by established providers that a country considers as adequately skilled (i.e., ‘experts’). Experts performing screening or reviewing cases are considered to improve reproducibility of VIA. Moreover, experts mentor trainees to accurately perform at least 50 VIA/SEVIA exams and ‘accurately’ identify and treat at least 5 precancerous lesions [9]. Within a few weeks of training and mentorship, the agreement between trainees and experts was high [9]. This agreement used the expert review as the reference standard [9], with no evaluation of accuracy of diagnosis, as obtaining cervical biopsies (or even cytology) in the Tanzanian setting was not possible at that time due to availability and costs.

In the study presented here, with histopathology and HPV status on all cervical images, we were able to evaluate providers’ reviews of cervical images in terms of clinical accuracy. This means that visual evaluations (overall and by providers’ experience level) were compared with known disease diagnosis of a cervical image. We found that accuracy of visual evaluations in this subset had a moderate sensitivity of 60.0% (95% CI 59.2 − 60.7) across all levels of provider experience- and training-related factors and that the impact of higher experience was weaker than expected. Providers with more experience gave fewer positive reviews to images with normal diagnosis, meaning that they achieved higher specificity, but they also gave fewer positive reviews to images with precancer diagnosis, meaning they were less sensitive as compared to less experienced providers. Among all experience and training variables, SEVIA training was the most discriminatory between providers’ experience level, although this alone did not translate into higher overall accuracy of providers who received SEVIA training. Of note, this was a one-time evaluation, thus the impact of training in accuracy of diagnosis (e.g., accuracy before/after SEVIA) could not be measured. An explanation for the small difference in accuracy and for the high level of agreement between trainees and experts as seen in Yeates et al [9] could be the rapid transfer of the basics of cervical visual evaluation. In fact, VIA training and positivity criteria are simpler than other methods, and trainer and trainee may require a reduced time interval to agree among themselves with little differences between experience levels. On the contrary, in colposcopy there are many criteria to diagnose a lesion and even experts do not agree [5,6,18]. As most apprenticeship-type programs are often based on the expert’s opinion, it is desirable to have quality assurance based on an objective reference standard measure. Despite the conventional reference standard of screening and diagnosis, colposcopy and biopsy respectively are themselves not objective [18], they still offer an external measure of quality. For instance, in this work, we defined reference standard using histopathology and HPV status. It would need to be assessed whether, for example, accuracy of visual impressions would increase if feedback to providers during the learning process was based on biopsy results.

This study has several strengths, including the use of histology combined with HPV status to define the reference diagnosis categories and the very large number of provider reviews (n = 24,300), which enabled stable estimation of image-level positivity across reference diagnosis categories and experience/training subgroups. As noted earlier, a full clinical evaluation of VIA was not the objective of this work, and several limitations should be considered in that context. The image set was intentionally pre-selected for high quality and enriched with precancer cases, which limits generalizability to routine screening settings where the spectrum of disease and image quality is more variable. Moreover, the use of single, static images viewed under timed conditions does not fully reflect real-world VIA practice, where providers can reposition, reapply acetic acid, and incorporate broader clinical information or other patient-associated factors that may influence performance. That said, clinical maneuvering and adaptive visualization during a standard unaided VIA exam are still much more limited than in a full colposcopic assessment. Because magnification could enhance visibility of acetowhite boundaries, our approach may overestimate routine field performance. The generalizability of our findings may also be influenced by the baseline HIV prevalence in Tanzania, which is higher than in the reference cohorts. Although the morphologic diagnostic criteria of precancerous lesions are the same regardless of HIV status, women living with HIV often develop more advanced and larger lesions than HIV-negative women [19,20]. Nevertheless, because we recruited providers from national CECAP facilities serving the general population rather than HIV clinics, this factor is unlikely to have impacted our performance estimates. Additionally, while this study focused on diagnostic interpretation, we did not evaluate the providers’ ability to determine eligibility for ablative therapy, which is a critical component of the visual assessment. Finally, experience and training measures were self-reported, which may introduce misclassification or recall bias in subgroup analyses, though this does not affect estimates of the overall performance of visual impressions.

Visual assessment of the cervix is inherently difficult, and our results underscore the need to rigorously evaluate the accuracy of visual programs, including those supported by mHealth interventions. While these approaches offer opportunities for training, supervision, and quality assurance, their impact on true diagnostic accuracy appears limited. Our findings therefore have important implications for cervical cancer screening programs in LMICs, where VIA continues to be used as a primary screening method largely because of its low cost and ease of implementation. Despite these advantages, relying solely on VIA risks perpetuating gaps in sensitivity and missed opportunities for early detection. As more affordable HPV tests, self-sampling strategies, and decentralized testing platforms become available, implementation of HPV-based screening – recommended by WHO – should not be postponed. Ultimately, sustainable and scalable screening programs must balance feasibility with diagnostic accuracy, and HPV testing offers a more objective and reliable foundation for reducing cervical cancer burden in resource-constrained settings.

As Tanzania and other LMICs transition toward HPV-based primary screening [21], two critical challenges remain. First, current multi-day screening strategies often suffer from high rates of loss to follow-up between HPV testing and triage/treatment. The emergence of point-of-care or near-point-of-care HPV platforms will be essential to facilitate single-day models to minimize loss to follow-up. Second, in many national programs, VIA continues to be utilized as a triage method for HPV-positive women. Our findings regarding the inconsistent performance of visual interpretation—regardless of provider experience—underscore the risk of using subjective triage, which may compromise the high sensitivity gained from molecular testing. Furthermore, a critical limitation of any visual assessment is its inability to detect HPV-positive high-grade lesions that lack distinct morphological changes. If a lesion is anatomically occult to visual inspection, no amount of provider experience can overcome this barrier. This highlights the urgent need for more objective, reliable, and risk-based clinical management strategies to ensure that the shift to HPV screening achieves its full public health potential. Moving forward, the role of VIA may be to serve as artificial intelligence (AI)-supported triage tool following primary HPV testing [22]. When paired with HPV extended genotyping, AI-supported visual triage can help guide risk-based clinical management, particularly for lower-risk genotype groups where immediate treatment may not be indicated or possible [23,24]. The ultimate goal remains unchanged: to minimize visual and diagnostic misclassifications so that resource-limited programs can efficiently detect and treat the true disease precursor before it progresses to cervical cancer.

## Conclusions

In summary, while training and expert mentorship remain valuable for many aspects of clinical practice and program implementation, our findings suggest that experience alone does not guarantee improved clinical accuracy of visual evaluation when judged against virologic and histologic outcomes. Visual impressions of enlarged digital cervical images had only moderate sensitivity and the provider experience and training, as measured here, did not have a major impact on accuracy. This emphasizes the need for objective external quality measures, timely shift to HPV-based approaches, and cautious interpretation of concordance-based training evaluations.

## Supporting information

S1 Appendix Table A. Dataset used for the image-level analysis. Each figure represents the number of providers in a given experience/training category who labeled each image as positive among the total of 486 providers. Image reference diagnosis is given (sorted by: precancers, indeterminate, normals). Abbreviations: ID, identifier; SEVIA, smartphone-enhanced VIA; VIA, visual inspection with acetic acid. **Table B.** Average proportions of providers (and corresponding 95% CI) reading each image as positive/negative by precancer/normal diagnoses, separately by experience/training subgroups. Abbreviations: SEVIA, smartphone-enhanced VIA; VIA, visual inspection with acetic acid. **Checklist A.** PLOS’ questionnaire on Inclusivity in Global Research for Human subjects research.
(DOCX)

## Abbreviations

AI, artificial intelligence; ASC-US, atypical squamous cells of undetermined significance; AVIVA, APIN-developed VIA visual application; CECAP, cervical cancer prevention; CI, confidence interval; CIN, cervical intraepithelial neoplasia; DNA, deoxyribonucleic acid; eC3, electronic cervical cancer control; EGPAF, Elizabeth Glaser pediatric AIDS foundation; HIV, human immunodeficiency virus; HPV, human papillomavirus; HSIL, high-grade squamous intraepithelial lesion; LEEP, loop electrosurgical excision procedure; LLETZ, large loop exicision of the transformation zone; LMIC, low- and middle-income country; LSIL, low-grade squamous intraepithelial lesion; MDH, management and development for health; MOH, Ministry of Health; NCI, National Cancer Institute; SD, standard deviation; SEVIA: smartphone-enhanced VIA
STM, specimen transport medium; VIA: visual inspection with acetic acid
