# Supplementary material for: Challenging the assumption that experience- and training-related factors improve accuracy of cervical cancer screening image interpretation: Evidence from Tanzania
Source: PLOS Glob Public Health. 2026 Aug 10;6(8):e0007074. doi: 10.1371/journal.pgph.0007074 (PMC13456253; doi:10.1371/journal.pgph.0007074)
Supplement: Supplementary file 1 [file pgph.0007074.s001.docx]

**S1 Appendix - Supplement to:**

**CHALLENGING THE ASSUMPTION THAT EXPERIENCE- AND TRAINING-RELATED FACTORS IMPROVE ACCURACY OF CERVICAL CANCER SCREENING IMAGE INTERPRETATION: EVIDENCE FROM TANZANIA**

Table of Contents

[**Table A.** Dataset used for the image-level analysis.	2](#_Toc236159733)

[**Table B.** Average proportions of providers (and corresponding 95% CI) reading each image as positive/negative by precancer/normal diagnoses, separately by experience/training subgroups.	4](#_Toc236159734)

[**Checklist A.** PLOS’ questionnaire on Inclusivity in Global Research for Human subjects research.	5](#_Toc236159735)

## **Table A. Dataset used for the image-level analysis.**

Each figure represents the number of providers in a given experience/training category who labeled each image as positive among the total of 486 providers. Image reference diagnosis is given (sorted by: precancers, indeterminate, normals).

| **Image ID** | **Reference diagnosis** | **Experience/Training variables** | | | | | | | | | |
| --- | --- | --- | --- | --- | --- | --- | --- | --- | --- | --- | --- |
|  |  | **Years VIA experience** | | **N weekly VIA exams** | | **VIA refresher training** | | **SEVIA training** | | **Expert group** | |
|  |  | **Tertile 3** (n=150) | **Tertile 1-2** (n=334) | **Tertile 3** (n=159) | **Tertile 1-2** (n=318) | **Yes**  (n=237) | **No**  (n=244) | **Yes**  (n=63) | **No**  (n=422) | **Yes**  (n=20) | **No**  (n=466) |
| 2 | Precancer | 131 | 272 | 127 | 268 | 189 | 209 | 43 | 359 | 17 | 386 |
| 4 | Precancer | 53 | 162 | 69 | 142 | 101 | 114 | 20 | 196 | 4 | 212 |
| 6 | Precancer | 110 | 233 | 108 | 230 | 173 | 170 | 49 | 296 | 15 | 330 |
| 9 | Precancer | 90 | 192 | 92 | 186 | 147 | 134 | 29 | 254 | 12 | 271 |
| 11 | Precancer | 125 | 280 | 129 | 270 | 197 | 204 | 53 | 352 | 19 | 386 |
| 13 | Precancer | 138 | 315 | 153 | 293 | 220 | 230 | 55 | 399 | 17 | 438 |
| 14 | Precancer | 103 | 239 | 120 | 218 | 157 | 183 | 28 | 316 | 9 | 335 |
| 15 | Precancer | 71 | 183 | 93 | 159 | 127 | 126 | 24 | 231 | 7 | 248 |
| 16 | Precancer | 28 | 77 | 34 | 69 | 46 | 58 | 9 | 97 | 1 | 105 |
| 18 | Precancer | 16 | 56 | 28 | 43 | 41 | 30 | 4 | 67 | 0 | 72 |
| 20 | Precancer | 114 | 280 | 129 | 260 | 190 | 201 | 48 | 348 | 14 | 382 |
| 21 | Precancer | 36 | 104 | 47 | 91 | 63 | 77 | 8 | 134 | 3 | 139 |
| 22 | Precancer | 102 | 222 | 109 | 210 | 155 | 168 | 47 | 279 | 15 | 311 |
| 23 | Precancer | 49 | 165 | 74 | 137 | 94 | 120 | 16 | 200 | 5 | 211 |
| 24 | Precancer | 59 | 155 | 75 | 136 | 99 | 114 | 26 | 190 | 11 | 205 |
| 26 | Precancer | 76 | 203 | 95 | 180 | 124 | 154 | 26 | 255 | 7 | 274 |
| 27 | Precancer | 67 | 186 | 85 | 164 | 121 | 131 | 23 | 231 | 9 | 245 |
| 31 | Precancer | 90 | 188 | 99 | 177 | 139 | 137 | 44 | 235 | 14 | 266 |
| 34 | Precancer | 87 | 221 | 104 | 199 | 144 | 162 | 34 | 275 | 13 | 296 |
| 35 | Precancer | 74 | 191 | 88 | 173 | 126 | 138 | 27 | 240 | 7 | 260 |
| 39 | Precancer | 125 | 288 | 135 | 272 | 200 | 212 | 53 | 362 | 17 | 398 |
| 41 | Precancer | 114 | 260 | 130 | 238 | 178 | 194 | 43 | 333 | 11 | 365 |
| 43 | Precancer | 72 | 151 | 68 | 151 | 102 | 119 | 20 | 203 | 8 | 215 |
| 45 | Precancer | 90 | 229 | 106 | 208 | 147 | 171 | 30 | 291 | 8 | 313 |
| 49 | Precancer | 142 | 328 | 155 | 309 | 230 | 237 | 62 | 409 | 19 | 453 |
| 50 | Precancer | 43 | 156 | 62 | 133 | 81 | 117 | 16 | 183 | 3 | 197 |
| 1 | Indeterminate | 74 | 140 | 72 | 138 | 109 | 103 | 31 | 182 | 11 | 203 |
| 3 | Indeterminate | 83 | 201 | 96 | 184 | 137 | 146 | 23 | 262 | 7 | 279 |
| 8 | Indeterminate | 26 | 86 | 33 | 77 | 49 | 64 | 6 | 108 | 1 | 113 |
| 12 | Indeterminate | 10 | 34 | 19 | 25 | 21 | 24 | 4 | 41 | 2 | 43 |
| 17 | Indeterminate | 20 | 62 | 28 | 52 | 36 | 46 | 6 | 77 | 2 | 81 |
| 19 | Indeterminate | 83 | 185 | 91 | 173 | 119 | 147 | 22 | 246 | 8 | 261 |
| 25 | Indeterminate | 104 | 249 | 114 | 233 | 169 | 181 | 32 | 321 | 8 | 346 |
| 28 | Indeterminate | 14 | 53 | 17 | 49 | 31 | 35 | 3 | 63 | 0 | 67 |
| 32 | Indeterminate | 7 | 30 | 7 | 30 | 12 | 25 | 4 | 33 | 0 | 37 |
| 33 | Indeterminate | 81 | 200 | 89 | 189 | 128 | 150 | 34 | 249 | 8 | 275 |
| 36 | Indeterminate | 50 | 123 | 68 | 101 | 76 | 96 | 13 | 161 | 6 | 168 |
| 37 | Indeterminate | 34 | 113 | 49 | 96 | 53 | 93 | 10 | 139 | 2 | 147 |
| 38 | Indeterminate | 13 | 36 | 20 | 27 | 22 | 26 | 1 | 48 | 1 | 48 |
| 42 | Indeterminate | 22 | 96 | 31 | 85 | 54 | 64 | 8 | 112 | 2 | 118 |
| 44 | Indeterminate | 21 | 72 | 27 | 65 | 46 | 46 | 5 | 87 | 1 | 92 |
| 47 | Indeterminate | 14 | 34 | 9 | 38 | 17 | 30 | 4 | 44 | 0 | 48 |
| 5 | Negative | 62 | 167 | 67 | 159 | 95 | 133 | 11 | 218 | 3 | 227 |
| 7 | Negative | 99 | 212 | 104 | 201 | 145 | 166 | 31 | 282 | 12 | 301 |
| 10 | Negative | 51 | 150 | 71 | 125 | 87 | 114 | 15 | 188 | 3 | 200 |
| 29 | Negative | 17 | 50 | 20 | 45 | 31 | 35 | 3 | 64 | 2 | 65 |
| 30 | Negative | 51 | 175 | 79 | 145 | 102 | 123 | 18 | 210 | 4 | 224 |
| 40 | Negative | 45 | 122 | 61 | 104 | 70 | 95 | 6 | 162 | 2 | 166 |
| 46 | Negative | 82 | 200 | 98 | 180 | 122 | 158 | 20 | 263 | 8 | 275 |
| 48 | Negative | 35 | 112 | 47 | 98 | 65 | 81 | 4 | 144 | 1 | 147 |

Abbreviations: ID, identifier; SEVIA, smartphone-enhanced VIA; VIA, visual inspection with acetic acid.

## **Table B. Average proportions of providers (and corresponding 95% CI) reading each image as positive/negative by precancer/normal diagnoses, separately by experience/training subgroups.**

|  | **Precancer diagnosis** | | **Normal diagnosis** | |
| --- | --- | --- | --- | --- |
| Providers’ reviews | **Years of VIA experience** | | | |
|  | ≥5 years | <5 years | ≥5 years | <5 years |
| Positive | 56.5 (55.2-57.9) | 61.4 (60.5-62.4) | 36.8 (34.3-39.4) | 44.5 (42.7-46.3) |
| Negative | 37.7 (36.3-39.0) | 34.4 (33.5-35.3) | 58.3 (55.7-61.0) | 52.8 (51.0-54.6) |
| Providers’ reviews | **Number of VIA exams performed weekly** | | | |
|  | ≥12 exams/week | <12 exams/week | ≥12 exams/week | <12 exams/week |
| Positive | 60.8 (59.5-62.2) | 59.5 (58.5-60.4) | 43.0 (40.4-45.6) | 41.5 (39.7-43.4) |
| Negative | 35.1 (33.8-36.4) | 35.6 (34.7-36.5) | 54.2 (51.6-56.8) | 54.8 (53.0-56.7) |
| Providers’ reviews | **Refresher training** | | | |
|  | Yes | No | Yes | No |
| Positive | 58.3 (57.2-59.4) | 61.6 (60.6-62.7) | 37.8 (35.7-39.9) | 46.4 (44.3-48.5) |
| Negative | 37.1 (36.0-38.2) | 33.7 (32.6-34.8) | 59.0 (56.8-61.1) | 50.1 (48.0-52.2) |
| Providers’ reviews | **SEVIA training** | | | |
|  | Yes | No | Yes | No |
| Positive | 51.1 (49.0-53.2) | 61.4 (60.6-62.2) | 21.4 (18.1-24.8) | 45.3 (43.8-46.9) |
| Negative | 43.0 (40.9-45.1) | 34.2 (33.4-35.0) | 74.8 (71.2-78.4) | 51.3 (49.7-52.9) |
| Providers’ reviews | **Expert group** | | | |
|  | Yes | No | Yes | No |
| Positive | 51.0 (47.4-54.6) | 60.4 (59.6-61.1) | 21.9 (16.1-27.7) | 43.1 (41.5-44.6) |
| Negative | 43.8 (40.3-47.4) | 35.0 (34.3-35.8) | 75.6 (69.5-81.8) | 53.5 (52.0-55.1) |

Abbreviations: SEVIA, smartphone-enhanced VIA; VIA, visual inspection with acetic acid.

## **Checklist A. PLOS’ questionnaire on Inclusivity in Global Research for Human subjects research.**

- Did you obtain written informed consent from a representative of the local community or region before the research took place? How did you establish who speaks for the community? Details of written informed consent obtained from study participants should be reported separately in the Methods section of your manuscript.

*Prior to study implementation, permission to conduct the study was obtained through the Tanzania national Cervical Cancer Prevention (CECAP) program in collaboration with the Ministry of Health and its implementation partners, Elizabeth Glaser Pediatric AIDS Foundation (EGPAF) and Management and Development for Health (MDH). The study was conducted during routine provider training and supervision sessions organized by the Ministry of Health with support from EGPAF and MDH. These institutions coordinate the national cervical cancer screening program and oversee provider training activities across regions. The Ministry of Health and its implementation partners therefore served as the appropriate institutional representatives for the cervical cancer screening program and participating providers. Study procedures and logistics were discussed with key Ministry of Health program leads and partner organizations prior to implementation. Ethical approval was subsequently obtained from the Tanzania National Institute for Medical Research (NIMR) and Kilimanjaro Christian Medical University College (KCMUCo), which review research conducted within the Tanzanian health system. Written informed consent was obtained from all participating health care providers before participation in the image review and questionnaire activities, as described in the Methods section.*

- How did members of the local community provide input on the aims of the research investigation, its methodology, and its anticipated outcome(s)?

*Input from local stakeholders was incorporated during the development of the study through collaboration with the Tanzania Ministry of Health CECAP program and its implementation partners (EGPAF and MDH). EGPAF and MDH are local implementing partners in the regions where the study took place. The study concept and procedures were discussed with program leaders and training facilitators to ensure that the evaluation aligned with ongoing national cervical cancer screening activities and could be implemented within routine provider training sessions.*

- When engaging with the local community, how did you ensure that the informed consent documents and other materials could be understood by local stakeholders?

*To ensure that study materials were understandable to local stakeholders, all consent procedures, study instructions, and image review activities were conducted in Swahili, the primary language used in clinical training and program activities in Tanzania. Consent forms and study materials were translated into Swahili and presented during supervised training sessions so that participants could ask questions and seek clarification before providing consent. This approach helped ensure that all participating providers clearly understood the purpose of the study and their role in the image review exercise.*

- Will the findings of the research be made available in an understandable format to stakeholders in the community where the study was conducted (e.g. via a presentation, summary report, copies of publications, etc.)? Please provide details of how this will be achieved.

*Study findings will be shared with key stakeholders involved in the Tanzania national CECAP program, including the Ministry of Health and implementation partners (EGPAF and MDH). Results will be presented during program meetings, including the Technical working group of the CECAP program, training sessions, and stakeholder discussions to support ongoing efforts to strengthen cervical cancer screening and quality assurance activities.*
